# Upon Rejection: Psychiatric Emergencies of Failed Asylum Seekers

**DOI:** 10.3390/ijerph15071498

**Published:** 2018-07-16

**Authors:** Georgios Schoretsanitis, Dinesh Bhugra, Sarah Eisenhardt, Meret E. Ricklin, David S. Srivastava, Aristomenis Exadaktylos, Sebastian Walther

**Affiliations:** 1 University Hospital of Psychiatry, 3008 Bern, Switzerland; saraheisenhardt11@googlemail.com (S.E.); sebastian.walther@upd.unibe.ch (S.W.); 2 Department of Psychiatry, Psychotherapy and Psychosomatics, and JARA-Translational Brain Medicine, RWTH Aachen University, 52074 Aachen, Germany; 3 Institute of Psychiatry, King’s College London, De Crespigny Park, London SE5 8AF, UK; dinesh.bhugra@kcl.ac.uk; 4 Department of Emergency Medicine, Inselspital, University Hospital Bern, Freiburgstrasse, 3010 Bern, Switzerland; meret.ricklin@gmail.com (M.E.R.); DavidShiva.Srivastava@insel.ch (D.S.S.); Aristomenis.Exadaktylos@insel.ch (A.E.)

**Keywords:** failed asylum seekers, psychiatric emergency services, psychiatric hospitalisation, acute stress

## Abstract

*Background:* The status of a refugee or asylum seeker is only recognised after legal processes. The uncertainty of these procedures or the rejection itself may severely impact mental well-being. *Methods:* We surveyed the patterns of psychiatric services used by patients whose applications for asylum had been rejected. In a retrospective investigation of admissions to the University Emergency Department in Bern, Switzerland between 1 March 2012 and 28 February 2017, we studied patients receiving a psychiatric consultation after their applications had been rejected. The primary endpoint was based on the comparison of these individuals with controls who were asylum seekers with pending asylum applications using the Mann-Whitney *U* test and the chi-square test (χ^2^) with a significance level of 0.05. *Results:* Thirty-eight cases were identified. There were more men than women and the mean age was 30.08 ± 9.62 years. Patients predominantly presented as walk-in patients (*n* = 16, 42.1%), most frequently due to suicidal ideation (*n* = 16, 42.1%). Stress-related disorders were the most common diagnosis (*n* = 29, 76.3%) and patients were mainly referred to inpatient treatment (*n* = 28, 73.7%). Patients with rejected applications were less likely to be living in reception centres than patients with a pending application (χ^2^ = 17.98, *p* < 0.001). *Conclusion*: The profile of asylum seekers whose applications had been rejected reflects individuals with high-stress levels, potentially aggravated by the negative asylum decision.

## 1. Introduction

Immigrants and asylum seekers comprise a highly vulnerable group exposed to factors that may increase their risk of developing mental health problems [1,2]. Over the past few years, there has been an extensive study of factors mediating the negative effects of migration on mental health. These factors are often classified as pre-, peri- or post-migration adversities [3,4,5]. Those individuals seeking refuge and/or asylum are defined by law. It has been shown that asylum seekers comprise a subgroup of immigrants requiring increasing attention [6,7,8]. The asylum process invariably includes the reception, registration, and examination of the application. Depending on the legislation of the host country, persons are allocated in reception centres during this procedure. In fact, asylum seekers are exposed to specific risk factors that relate directly to the asylum process—including insecurity, stress and the fear of involuntary repatriation [7,9,10], which may explain why the whole process of asylum determination may aggravate the severity of claimants’ symptoms [11]. Furthermore, it is not surprising that the length of the procedure itself may correlate with the prevalence of psychiatric symptoms [6,12], whereas detention in reception centres poses an additional challenge due to the limitations placed on the claimants’ freedoms. Moreover, the application outcome may amplify precedent psychopathology or trigger new psychiatric symptoms [7,8,13,14,15]. The claimants may suffer additional stress if they are due to be expelled [8,16,17]. Data are available on the use of mental health care services by asylum seekers and these results reflect valuable aspects of claimants’ needs [18,19,20,21]. As an increasing number of these patients require mental health services, this evidence may be instrumental in developing strategies to improve psychiatric services. Claimants may often have difficulties in accessing treatment [22]. The situation might be further aggravated by cultural and language barriers [4,23]. A previous study has investigated the mental health of rejected asylum seekers compared to groups of claimants with a pending or approved residence permit in Switzerland based on interviews and standardized questionnaires [14]. Nevertheless, there are no specific data on the use of mental health care services by rejected asylum seekers because the available evidence does not separate these individuals from persons with applications in various stages of the asylum process [24,25]. This methodological issue is of crucial importance as the asylum process may last for years and asylum seekers are a widely heterogeneous group with respect to psychopathology and the prevalence of mental disorders [7,10,19,26]. Likewise, the only available specified service for immigrants in Canton Bern is aimed at mental health care for all types of immigrants regardless of the asylum process phase; further, all persons during the asylum process are entitled to health insurance coverage with some restrictions.

The debate on the needs of rejected asylum seekers is not restricted to psychiatry as it involves other health care fields [27]. Therefore, it is helpful to assess psychiatric emergencies in failed asylum seekers separately from other emergencies. The increase in the numbers of asylum applications, in turn, makes it easier to collect large-scale data and, thus, to investigate the specific needs of failed asylum seekers. The main aim of the current study was to provide descriptive data on the use of psychiatric emergency services by failed asylum seekers. We also compared categorical outcomes between patients with failed and pending applications, in order to identify factors that may distinguish these two groups of patients. This control group (asylum seekers with pending application) was considered a better option than a group of immigrants with warranted residence or the population because it should yield smaller social and cultural differences from the rejected asylum-seeking group. We hypothesised that deleterious effects would be associated with the negative decision, therefore leading to more severe symptoms in the rejected patients.

## 2. Methods

This descriptive study included retrospective data from adult patients (age ≥18 years) admitted to the University Emergency Department (Universitäres Notfallzentrum, UNZ, Bern, Switzerland) for a psychiatric consultation (during an interdisciplinary medical examination or a specific psychiatric evaluation) between 1 March 2012 and 28 February 2017. The UNZ provides 24 h/day psychiatric emergency services and it is responsible for emergency mental health care in Canton Bern, Switzerland (with a population of over 1,000,000 inhabitants) including asylum centres in this catchment area. Consulting clinicians are mainly resident doctors under the supervision of senior doctors. The screening process of the medical records is described in detail in a previous paper [28].

First, we reviewed electronic medical records to detect patients with a migration background (i.e., non-Swiss nationality), which led to 1697 records. Second, we reviewed records in order to identify patients with an asylum application, which yielded 237 records. Lastly, we separated the records of patients subject to deportation (failed applications). Apart from the sociodemographic characteristics (age, gender, nationality, marital status, children, relatives in Switzerland), we were able to extract the following clinical data: the pathway to care (health professionals involved in the referral), the reason for presentation/referral, the ICD-10 diagnosis set by the consulting person, the referral outcome, the presence of an interpreter during the consultation and contact with psychiatric services prior to the recorded (current) consultation.

The pathway to care was classified as follows: walk-in, general practitioner or another medical doctor, ambulance, police, or reception centres. The reasons for presentation/referral were grouped as follows: suicidal thoughts, attempted suicide, auto-aggressive behaviour, aggression, depressive symptoms, sleep disorders, acute stress, somatic complaints, psychotic symptoms, psychosocial problems and use of medication. Diagnoses (ICD-10) were classified in accordance with diagnostic categories (F10–19, 20–29, 30–39, 40–49, F60–69, F90–99). The possible referral outcomes were admittance to a psychiatric clinic (voluntary or compulsory) or discharge home. For patients who visited the UNZ more than once, only the most recent registration was considered; six patients had two registrations, while there were three registrations for two other persons. The asylum decision predated all registrations for the persons being consulted more than once. Rudimentary case files or incomplete medical records were excluded from the analysis (*n* = 46).

The health-related patient data were exported anonymously for analysis. Only one author (MER) had access to the information that could identify the patient. None of the authors were potentially an attending physician of the patients involved. Retrospective analysis of data for this study was in accordance with the local regulatory authority and with the Declaration of Helsinki. No informed consent was necessary for this case note type of study. The protocol was approved by the Ethics Committee of Canton Bern (Project-ID Number 2018-00198).

### Statistical Analysis

The data were summarised using descriptive statistics. Comparisons were conducted between persons with pending and failed asylum application. The Mann-Whitney *U* test (M-W-*U*) was used for age and gender distributions, and the chi-square test (χ^2^) for categorical variables. When the latter test was not possible due to the small number of cases, Fisher’s exact test was conducted. A significance level of 0.05 was used for all comparisons. Referral outcome was transformed to a dichotomous variable; ‘0’ was used for cases of non-compulsory treatment consisting of discharge home and voluntary admissions in the clinic, whereas ‘1’ encoded compulsory (inpatient) treatment. This analysis was repeated using the type of treatment, as well as with out-vs-inpatient as a dummy variable. All statistical analyses were carried out using IBM SPSS Statistics, version 22.0 (IBM GmbH, Ehningen, Germany).

## 3. Results

We were able to identify 38 records of patients (15 women and 23 men) with a rejected asylum application (Table 1). In 31 cases, the patients were informed of the application outcome within 2 weeks prior to the psychiatric consultation (81.6%); 3 patients had previously presented at least once at UNZ shortly after the outcome of the application (7.89%); 3 patients had presented due to other triggers (7.89%); 1 patient presented shortly before the deportation day (2.6%). The mean age was 30.08 ± 9.62 years. In terms of origin, the individuals came from a range of countries; the most common countries of origin were Russia (*n* = 5), Eritrea (*n* = 3), Sri Lanka (*n* = 3), Syria (*n* = 3) and Vietnam (*n* = 3) (Table 2). One-third of the patients were single (*n* = 13, 34.2%), whereas 10 were married (26.3%). Seventeen patients (44.7%) did not have children, while 10 patients (26.3%) did (in 7 cases, the children lived in Switzerland). For 11 patients (28.9%), no information regarding children was available. Almost half of the patients (*n* = 18, 47%) had no relatives in Switzerland (see Table 1).

Patients predominantly came as walk-in patients (*n* = 16, 42.1%), whereas 10 (26.3%) were referred to the UNZ by the ambulance service (Table 3). Patients were most frequently referred due to suicidal thoughts (*n* = 16, 42.1%). Stress-related disorders (F40–49) comprised the most common diagnoses (*n* = 29, 76.3%). Consultants diagnosed an adjustment disorder (F43.2) in 14 cases (36.8%) and an acute stress reaction (F43.0) in 8 cases (21.1%).

The researchers found that 7 of the 8 patients with a diagnosis of acute stress reaction were informed about the negative asylum decision shortly before referral. Twenty-four patients had prior contact with psychiatric services (63.2%). The consultation was aided by an interpreting service in 9 cases (23.7%) or by the presence of a person who could speak the patient’s native language (family member or friend of the patient) in 5 cases (13.2%), which was not required or possible for the majority of the patients (*n* = 23, 60.5%).

Regarding the referral outcomes, patients were mainly treated as inpatients (*n* = 28, 73.7%); in 12 of these cases (31.6%), treatment was voluntary. A detailed description of sociodemographic and clinical characteristics of the patients with a pending application (control group) is provided elsewhere [28]. No association was detected between interpretation (by interpreting service or person from the patient’s environment) and compulsory treatment (*p* = 0.58 for Fisher exact test).

No differences for age and gender distribution were reported between asylum seekers with failed and pending applications (*p* > 0.05 for M-W-*U* for both comparisons). Nevertheless, there was a non-significant trend for gender, with proportionally more women in the group of patients with a rejected application than in the group of patients with a pending application (*p* = 0.092). Groups did not differ in the basic demographics, e.g., being single, having children or relatives in Switzerland (*p* > 0.05 in both cases). Nor did the groups differ in terms of prior contact with mental health care or in the presence of an interpreter during the consultation (*p* > 0.05 in both cases). A lower proportion of failed asylum seekers resided in reception centres than claimants with pending applications (χ^2^ = 17.98, df = 2, *p* < 0.001). Regarding referral reasons, the rates of suicidal ideation and attempted suicide were higher in patients with a rejected application rather than with a pending application, whereas aggressive behavior was more frequent in patients with a pending application; nevertheless, these differences did not reach statistical significance (χ^2^ = 15.97, df = 11, *p* = 0.14). Although the ambulance was more frequently involved in the referral process for failed claimants, this trend was not significant (χ^2^ = 7.77, df = 4, *p* = 0.1). In the same comparison, the police were more frequently involved for patients with pending than with rejected application. Regarding diagnoses, the proportion of individuals receiving a stress-related diagnosis (F40–49) was higher for rejected asylum seekers than for the control group (χ^2^ = 17.59, df = 7, *p* = 0.014). No differences were reported for referral outcomes, assignment to compulsory treatment or to inpatient setting (*p* > 0.05 for all three comparisons), although the inpatient admission rates were numerically higher in patients with a rejected application than a pending application (73.7% vs. 59.7%).

## 4. Discussion

Our retrospective study assessed psychiatric emergencies as reflected in patterns of use of mental health care services by persons with a failed asylum application. This subgroup of asylum seekers has not been widely studied, as they are commonly considered together with other individuals with asylum applications in various phases of the determination process [20].

Asylum seekers with rejected applications were predominantly young, male and single. In almost 90% of the cases, the negative refugee decision triggered severe psychic symptoms that led to psychiatric consultations at UNZ. The major pathway to the mental health care system was self-referrals (for 40.5% of patients); patients presented themselves to the facility most frequently for reported suicidal thoughts (40% of cases) followed by an evaluation after a suicide attempt (in 13.2%). In a Danish study with 61% of rejected asylum seekers, researchers reported suicidal ideation as the most common reason for consultation [20]. One out of three failed asylum seekers was given a diagnosis of adjustment disorders, which is in alignment with previous data reporting high-stress levels and depressive symptoms for patients with rejected applications [7,10,22].

Another finding deserving particular attention is the high percentage of patients treated as inpatients, which may relate to the lack of outpatient or social resources for these patients [29]. Alternatively, this finding might be related to the alarmingly high levels of stress and psychological symptoms suffered by rejected asylum seekers [22,24]. On the other hand, for involuntary inpatient treatment, there were no differences between persons with rejected and pending applications.

Moreover, the proportion of individuals diagnosed with a stress-related disorder was higher for the failed asylum seekers than for the control group of asylum seekers with pending decisions. In fact, the rates of acute stress reactions were 3 times higher for the failed asylum seekers than for the control group of asylum seekers with pending decisions (21% vs. 7.6%). Note that 7 out of 8 rejected asylum seekers receiving the diagnosis of acute stress reaction were referred shortly (within 24 h) after the negative asylum decision. Because severe symptoms have been reported in many claimants even during the determination process [28], we posit that the decision may have aggravated pre-existing symptoms, rather than triggering them. Here, we have to consider the possible adverse effects of the pending status as well. Ambulances were used for one-fourth of these patients–indicating that the symptoms were severe in the acute phase, but the differences were not statistically significant. Although suicide attempts were more frequent in failed claimants than in claimants with pending decisions, this difference also did not reach significance.

There were significant differences in the type of residence between rejected asylum seekers and persons with a pending application: the proportion of failed asylum seekers residing in reception centres was lower than for claimants with a pending application. This result may have occurred because claimants with a pending application are forced to stay in reception centres for long periods. Nevertheless, data for the duration of stay in Switzerland were not consistently available in the medical records, so we cannot control for this confounding variable.

The number of consultations supported by the presence of an interpreting service (n = 9, 24.3%) was low compared to previous hospital data [20], but this may be due to bilingualism in Canton Bern, where German and French are the official languages. A group of immigrants came from French-speaking North African and Arabic countries and, therefore, French may offer a common language for them when contacting health care services. The association between interpretation (by interpreting service or person from the patient’s environment) and compulsory treatment for patients with a rejected application was not significant; this may also result from the small size of the group.

Our study has some major limitations that need to be remembered when interpreting the findings. This retrospective study was based on case notes. These case notes are of variable quality and may be unreliable. Thus, some patients were excluded due to incomplete information. The selection of the time period was rather arbitrary and relates to the availability of the electronic records. Parameters, such as the previous socio-economic status in the home country, the duration of stay in Switzerland before presentation or decision, symptom duration and severity assessed with established questionnaires, housing type (for the persons not residing in reception centres), and the reasoning for the organization of interpreting services were not available and therefore could not be considered. Moreover, no data were provided on the length of the asylum procedure before the decision. Nevertheless, these data seem to be crucial and their inclusion in the analysis could have provided valuable insight [30]. When multiple registrations per patient were available, we included the most recent. This highlights the need for follow-up data, which are widely lacking. Further, information regarding the duration or outcome of the inpatient treatment (for the patients referred to inpatient setting) was not available. Lastly, given the naturalistic design of this study, the control group was not matched for demographic or clinical characteristics with the group of asylum seekers.

## 5. Conclusions

Our observations indicate that persons with rejected applications entering mental health care facilities are relatively likely to suffer from severe psychiatric symptoms such as suicidal ideation as well as high levels of stress; under these circumstances, the asylum rejection decision may trigger additional acute stress, leading to psychiatric emergencies and consultations. These individuals are invariably treated as inpatients, which may reflect high levels of psychological symptoms and/or the scarce social resources of these patients. Although findings indicate that failed claimants exhibit more acute symptoms, such as suicidal ideation or suicide attempts, than patients with pending status, these differences were not statistically significant. One possibility is that there is a continuum of severe symptoms during the determination process, which may be aggravated by the rejection decision. Providers should be aware of the particular psychopathology patterns in these highly vulnerable individuals. Given the limited social resources of these patients, inpatient treatment may be more appropriate for these patients than for other patient groups. Finally, an intensive support might prevent symptom aggravation during the final phase of the asylum procedure, i.e., shortly before and after the application outcome is announced.

## Figures and Tables

**Table 1:** The sociodemographic characteristics of patients with rejected (*n* = 38) and pending application (*n* = 119).

Variable	Rejected Application	Pending Application	
Females (%)	15 (39.5)	30 (25.2)	*p* = 0.092
Age (SD)	30.08 (9.62)	29.88 (9.13)	*p* = 0.964
Marital status (%)	*n* (%)	*n* (%)	
Never been married	13 (34.2)	67 (56.3)	χ^2^ = 7.518, df = 5, *p* = 0.185
Widowed and Divorced	5 (13.2)	9 (7.5)	
Married (Spouse in CH)	7 (18.4)	13 (10.9)	
Married (Spouse abroad)	3 (7.9)	11 (9.2)	
Unknown	10 (26.3)	19 (16.0)	
Children (%)	*n* (%)	*n* (%)	
No	17 (44.7)	72 (60.5)	χ^2^ = 4.008, df = 5, *p* = 0.261
Yes (Children in CH)	7 (18.4)	18 (15.1)	
Yes (Children abroad)	3 (7.9)	10 (8.4)	
Unknown	11 (28.9)	19 (16.0)	
At least one relative in CH, not partner (%)	*n* (%)	*n* (%)	
No	18 (47.4)	66 (55.5)	χ^2^ = 2.075, df = 2, *p* = 0.354
Yes	10 (26.3)	34 (28.6)	
Unknown	10 (26.3)	19 (16.0)	
*Residing in reception center (%)*	*n* (%)	*n* (%)	
Yes	14 (36.8) *	85 (71.4)	* χ^2^ = 17.98, df = 2, *p* < 0.001
No	23 (60.5) *	28 (23.5)	
Unknown	1 (2.6)	6 (5)	

*: Statistically significant. Age provided in years, CH: Switzerland.

**Table 2:** The regions of the origin of persons with a rejected application (*n* = 38).

Regions of Origin (%)	
Middle East	7 (18.4)
Eastern Europe	8 (21.1)
Sub-Saharan Africa	11 (28.9)
Northwestern Africa	4 (10.5)
Central-South Asia	8 (21.1)

**Table 3:** The clinical characteristics of rejected (*n* = 38) and pending applications (*n* = 119).

Variable	Rejected Application	Pending Application	
Pathway to UNZ Emergency Department	*n* (%)	*n* (%)	
Walk-in	16 (42.1)	52 (43.7)	χ^2^ = 7.771, df = 4, *p* = 0.1
General practitioner (or other MDs)	2 (5.3)	16 (13.4)	
Ambulance	10 (26.3)	13 (10.9)	
Police	8 (21.1)	35 (29.4)	
Reception center	2 (5.3)	3 (2.5)	
Referral reasons	*n* (%)	*n* (%)	
Suicidal ideation	16 (42.1)	30 (25.2)	χ^2^ = 15.968, df = 11, *p* = 0.142
Suicide attempt	5 (13.2)	3 (2.5)	
Auto-aggressive behavior	3 (7.9)	7 (5.9)	
Aggressive behavior	3 (7.9)	25 (21.0)	
Psychotic symptoms	2 (5.3)	11 (9.2)	
Depressive symptoms	2 (5.3)	10 (8.4)	
Sleep disorders	2 (5.3)	8 (6.7)	
Acute stress	3 (7.9)	9 (7.6)	
Somatic complaints	1 (2.6)	10 (8.4)	
Psychosocial problems	1 (2.6)	2 (1.7)	
Medication acquisition	0 (0)	2 (1.7)	
Other	0 (0)	2 (1.7)	
ICD diagnoses	*n* (%)	*n* (%)	
Disorders due to substance use (F10–19)	2 (5.3)	14 (11.8)	* χ^2^ = 17.59, df = 7, *p* = 0.014
Schizophrenia spectrum disorders (F20–29)	2 (5.3)	22 (18.5)	
Affective disorders (F30–F39)	3 (7.9)	17 (14.3)	
Stress-related disorders (F40–F49)	29 (76.3) *	58 (49.6)	
Personality disorders (F60–69)	0 (0)	6 (5.0)	
Others	2 (5.3)	1 (0.8)	
Referral outcome	*n* (%)	*n* (%)	
Discharged home	4 (10.5)	32 (26.9)	χ^2^ = 4.412, df = 3, *p* = 0.22
Discharged home with outpatient treatment	6 (15.8)	16 (13.4)	
Voluntary admission	16 (42.1)	42 (35.3)	
Compulsory admission	12 (31.6)	29 (24.4)	

*: Statistically significant. ICD: International Classification of Diseases; MD: medical doctor; UNZ: University Emergency Department.
